# Comparison of Silane Heat Treatment by Laser and Various Surface Treatments on Microtensile Bond Strength of Composite Resin/Lithium Disilicate

**DOI:** 10.3390/ma14247808

**Published:** 2021-12-16

**Authors:** Goknil Ergun-Kunt, Rafat Sasany, Mehmet Faruk Koca, Mutlu Özcan

**Affiliations:** 1 Department of Prosthodontics, Faculty of Dentistry, University of Ondokuz Mayıs, Samsun 55420, Turkey; gergun@omu.edu.tr; 2 Independent Researcher, Samsun 55020, Turkey; 3 Department of Periodontology, Faculty of Dentistry, University of Ondokuz Mayıs, Samsun 55420, Turkey; mfarukkoca@yahoo.com; 4 Center of Dental Medicine, Division of Dental Biomaterials, Clinic for Reconstructive Dentistry, University of Zurich, 8032 Zurich, Switzerland; mutluozcan@hotmail.com

**Keywords:** adhesion, bond strength, ceramic, Er:YAG laser, heat treatment, silane

## Abstract

In the current study, we evaluated the effects of heat treatment (by Er:YAG or furnace) and various surface treatments on the microtensile bond strength (μTBS) of silanized lithium disilicate ceramic. Seventy lithium disilicate (IPS e. max Press; Ivoclar Vivadent) and composite resin (Tetric N-Ceram; Ivoclar Vivadent) blocks were made and distributed into seven groups (n = 10) at random: S: silanization alone; ALS: airborne particle abrasion (APA) and silanization; SC: APA modified with silica and silanization; SHT1: silanization and heat treatment by Er:YAG; SHT2: silanization and heat treatment performed in the furnace (100 °C, 1 min); HF: etching with HF; and HFS: etching with HF and silanization. Every ceramic specimen was cemented to a composite resin block after surface treatment. Cemented specimens were embedded into acrylic resin and were tested with the μTBS test. Data were analyzed using one-way ANOVA and Tamhane T2 tests (α = 0.05). The SHT1 group had the highest bond of strength compared to the other groups (27.46 MPa). The ALS group had the lowest strength of the groups (15.56 MPa). Between SHT2 and HFS (*p* = 1), the comparison of the mean µTBS values showed no significant differences. It was concluded that silane heat treatment increased the resin composite–ceramic bond strength; however, within the terms of μTBS, the Er:YAG laser treatment was more successful than other surface treatment applications.

## 1. Introduction

Aesthetics is one of the most critical characteristics in dentistry that has significantly increased the usage of all-ceramic systems [1,2,3,4,5]. All-ceramic materials for the manufacture of fixed dental prostheses play an essential role in rehabilitation. Lithium disilicate glass-ceramics are an alternative to traditional materials and have excellent aesthetic and mechanical properties [6,7,8,9,10,11]. Resin composite is widely used in definitive prostheses and temporary veneers due to its stability, excellent mechanical properties, and desirable aesthetics. Temporary veneers are also fundamental materials used in prosthetic rehabilitation and have essential biological, aesthetic, and practical functions [12,13]. Providing a reliable, permanent bond between the all-ceramic restoration and resin composite is essential for long-term clinical success. In addition, it is essential in terms of providing a balanced distribution of occlusal contacts [14,15]. The composite–ceramic bond is dependent on the adhesion of the composite resins to the ceramic surface treatment used, and these current processes are based on micromechanical and chemical bonding [16,17]. The ceramic surfaces are prepared in a variety of ways [18], such as hydrofluoric acid etching, (HF) [16,17,19,20,21,22,23,24,25,26,27] silica coating [19], abrasion by airborne aluminum oxide particles [28], and laser irradiation [29]. These methods are used to increase surface roughness and bond strength. Air abrasion may improve ceramic surface roughness and wettability; however, it induces microcracks in the structure and leads to new fractures in the long term [28]. In addition, the intraoral method may be limited due to the unfavorable operating circumstances associated with alumina particles [28]. The chemical bond between ceramic and composite resin is mainly caused by silane coupling agents; by forming a siloxane bond in response to an increase in the surface energy of the ceramics and the wettability of the cement, silane coupling agents establish adhesion between the inorganic phase of the ceramic and the organic phase of the bonding agent applied to the ceramic surface, resulting in microscopic interactions between both parts [19]. Hydrofluoric acid (HF) etching is one of the most prevalent ways to efficiently bond resin to the surface of glass ceramics [16,19,20,21,22,23,24,25,26,27,28,29,30,31,32,33,34]. Cementation of glassy matrix ceramics following HF etching and silanization has been recommended in several studies [17,19,27]. By selectively dissolving the glassy ceramic matrix to create a rough surface, HF treatment improves the topography to favor micro-mechanical retention [11]. The bond affinity between fluoride and silicon is more significant than that between silicon and oxygen, explaining the mechanism. The acid selectively dissolves the glassy matrix of the lithium disilicate surface, exposing the crystalline structure responsible for resin cement micromechanical retention [11].

Nowadays, the laser technique is becoming more popular in the dentistry field. The Er:YAG laser wavelength is 2940 nm, which corresponds to the maximum of water absorption at the invisible spectrum [18,29]. Heat treatment may accelerate the silane, resulting in a long-lasting bond that strengthens the composite–ceramic link [3,5,13,19,27,28,29,30,31,32,33,34,35]. Water, alcohol, and additional particles are removed from the ceramic surface during heat treatment; the removal of water accelerates the completion of the silane/silica surface condensation reaction and increases the creation of covalent silane/silica bonds. Evaporation of chemicals that would ordinarily hydrogen bond to the silica surface, such as alcohol, increases the number of bond sites available for interaction with silane [32,33]. Some methods have been described for the heat treatment, and the most common ones include drying with warm air [3,32,33], rinsing with hot water [13,31], using a preheated furnace or hot air oven [5,19,32], and a combination of these methods [15,33].

After the primer for adhesion was introduced, the conventional shear or tensile bond tests increased [34,35]. With this procedure, the measured pressures are around 18–20 MPa [34,35]. It was challenging to identify bond strength values that differed significantly. Additionally, failures with lower bond strength (9 MPa) were common [34,35]. A novel tensile bond test with a low surface area indicated more adhesive failure at the bonded interface or higher bond strength than conventional bond tests with larger surface areas, which occasionally indicated cohesive failure within dentin at less than 5 MPa [34,35]. The µTBS test is now widely accepted as a versatile and reliable method of determining bond strength. Morphological and spectroscopic investigations demonstrated a greater discriminative capacity than the conventional macro-shear test in improving resin/dentin adhesion [36].

This study was conducted to compare the impact of different surface treatments and two methods of silan heat treatments (laser and furnace) on the bond strength between composite resin and lithium disilicate. According to the null hypothesis, silane heat treatment by Er:YAG laser could not improve the bond strengths enough to compare differing surface treatments between composite resin and lithium disilicate.

## 2. Materials and Experiments

The materials used in this study are listed in Table 1. 

Seventy square-shaped lithium disilicate (IPS e. max Press; Ivoclar Vivadent, Schaan, Liechtenstein) ingots (13 mm × 13 mm × 13 mm) were made using the lost-wax method, followed by ceramic ingots injected under high pressure at 925 °C for 20 min under a pressure of 4.5 bar in a furnace (Programat EP500, Ivoclar Vivadent, Schaan, Liechtenstein) [37] according to the manufacturer’s instructions. All specimens were mechanically polished with silicon carbide abrasives of 600, 800, and 1000 grit. To make a duplicate mold for the polymerization of composite blocks, ceramic blocks were inserted in polyvinyl siloxane impression material (Elite HD+; Zhermack, Badia Polesine, Italy). Composite resin (Tetric N-Ceram; Ivoclar Vivadent) layers up to 1.5 mm thick were placed into copy molds, then polymerized for 20 s with a light-emitting diode (LED) (Hilux LED MAX 1055; Benlioglu dental, Çankaya/Ankara, Turkey), yielding 70 composite resin blocks with the exact dimensions of the ceramic blocks.

All specimens were divided into seven groups:

- S group (silane): A silane coupling agent (Monobond S, Ivoclar Vivadent, Schaan, Liechtenstein) was applied to the surface specimens for 1 min and then air-dried for 30 s.
- SC group: Air abrasion was applied by 30 m aluminum oxide (Al_2_O_3_) particles modified with silica (Cojet Sand; Seefeld, Germany) at a pressure of 2.3 bar from a distance of 10 mm for 15 s (tribochemical silica coating). Then silane was applied.
- ALS (AL ± silane) group: Sandblasting was performed with 50 m Al_2_O_3_ particles (Korox 50; Bego Bremen, Germany) at a pressure of 0.3 MPa from a distance of 10 mm for 10 s [38]. Then silane was applied.
- SHT1 group (laser ± silane): Specimens were irradiated for 30 s with an Er:YAG laser using a non-contact head. The laser had a frequency of 20 Hz, a long pulse of 5 W, and a power of 250 mJ.
- SHT2 group (silane ± heat treatment): Silane was applied on the surface according to the same procedure and then heat treatment was performed in the furnace (100 °C, 1 min).
- HF group: The surface of the specimens was etched for 20s with 9.5 percent HF gel (Porcelain Etch; Ultradent Products Inc, South Jordan, UT, USA), washed for 1 min, and air-dried for 1 min.
- HFS group (HF ± silane): The acid-etching methodology was used. The technique was also followed by silanization.

The ceramic specimens were glued to composite resin (Tetric N-Ceram; Ivoclar Vivadent, Schaan, Liechtenstein) under a 500 g load after surface treatment. For 24 h, bonded specimens (ceramic-composite) were kept in dark containers filled with distilled water at 37 °C. All specimens were aged (thermal cycling 5000 cycles, 5–55 °C, dwell time: 30 s) before the microtensile bond strength test (µTBS) (Dentist Solubris Technica, Istanbul, Türkiye) [30].

The specimens were embedded in acrylic resin (Paladent; Heraeus Kulzer, Hanau, Germany and longitudinally sectioned at 1.3 mm intervals in both X and Y directions, perpendicular to the resin/ceramic interface, with a precision saw (Isomet 1000; Buehler, Illinois, ABD). Under a stereomicroscope (Leica S4 E; Leica Microsystems, Heerbrugg, Switzerland), the ceramic-composite microbars (661 mm) were examined and evaluated for crack formation.

### 2.1. Microtensile Bond Strength Test (µTBS)

Every specimen was bonded to a microtensile testing machine (Micro Tensile Tester; BISCO Dental Products, Richmond, VA, USA) with cyanoacrylate (Pattex; Henkel, Germany) and exposed to tensile force at a crosshead speed of 1.0 mm/min. µTBS was calculated using the load at fracture (P) and is expressed in MPa using the P/A formula (N/mm^2^). 

### 2.2. Failure Mode

A stereomicroscope (Kaps ENT SOM Microscope, Asslar, Germany) was used to examine the debonded surfaces of the specimens at a magnification of 20× to determine the forms of failure (adhesive, cohesive, or mixed). In this study, we evaluated five types of adhesive failure mode [39].

### 2.3. Scanning Electron Microscopi (SEM)

**Figure 1:**
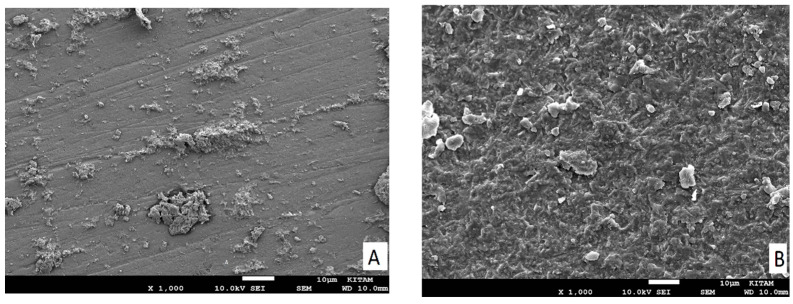

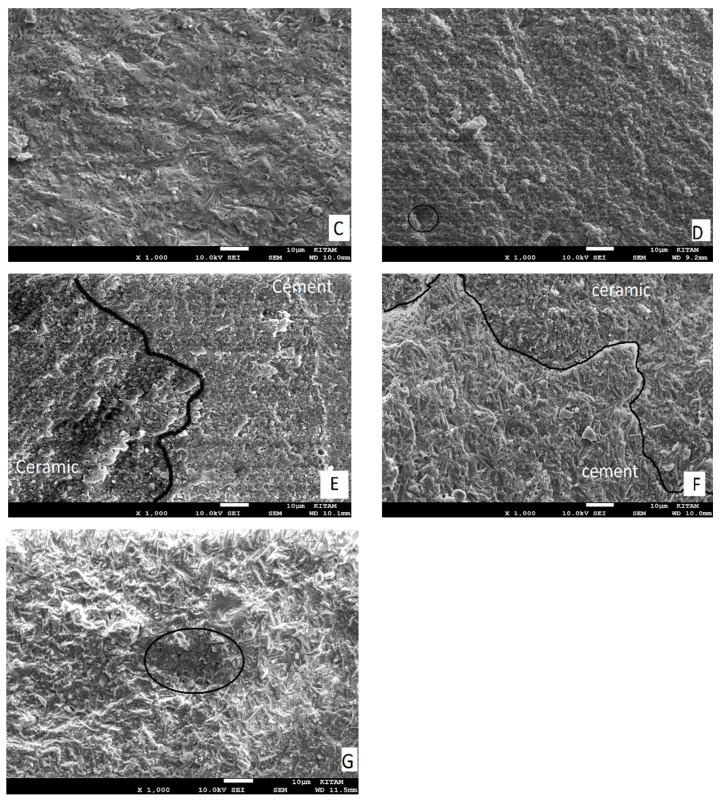
SEM micrographs of failure modes and the ceramic surface after treatment in a representative specimen: (**A**) Group S: adhesive failure (mode 1), where no trace of other substrate was found on the surface. (**B**) Group SC: adhesive failure (mode 4), where failure starts at the ceramic–adhesive interface as a corner flaw (lower left) and propagates through the adhesive resin. (**C**) Group ALS: adhesive failure (mode 5), where trace or other substrate was found on the surface. (**D**) Group SHT1) adhesive failure (Mode 2), the semicircular flaw is the crack origin; the adhesive resin is in the middle of the fracture surface. (**E**) Group SHT2: mixed failure mode, in a representative sample; lithium disilicate crystals are scarcely evident on the uncovered ceramic surface. (**F**) Group HF: mixed failure mode in a representative sample; the ceramic surface (black area) is partly covered with cement (white area), ceramic crystals are visible following the removal of the glassy phase. (**G**) Group SHF: adhesive failure (Mode 3), where the internal defect is the origin of the crack (black circle), the cement in the center of the fracture surface representing what qualifies as a failure.

A scanning electron microscope (SEM) (JSM-7001F; JEOL, Musashino, Akishima, Japan) was used to analyze one tested specimen from every group at 1000× magnification to assess surface characteristics of the performed treatment processes (Figure 1).

### 2.4. Statistical Analysis 

To evaluate significant changes across surface treatments, one-way ANOVA (IBM SPSS Statistics v21.0; IBM Corp, New York, Acmon, USA) and Tamhane T2 tests were performed. 0.05 was used as the significant level.

## 3. Results 

### 3.1. Microtensile Bond Strength Test (µTBS)

A one-way ANOVA analysis revealed statistically significant differences between the different surface treatment groups. (df = 6; F = 50.75; *p* < 0.001). The study groups’ mean, minimum, and maximum TBS values and standard deviations are shown in the list in Table 2. Group SHT1 had the highest mean bond strength (27.84 MPa), which was substantially different (*p* < 0.001) from that of the other groups. Group ALS had the lowest mean bond strength (15.62 MPa), which was likewise substantially different (*p* < 0.001) from that of the other groups, excluding the S group. No statistically significant difference (*p* = 1) was discovered between groups SHT2 and HFS, or between groups S and SC, according to the Tamhane T2 test results (Table 3).

### 3.2. Failure Mode 

Both mixed and adhesive-mode failures were discovered when the specimens were debonded. This study also observed five types of adhesive failure mode: 1: adhesive failure separation at the ceramic-cement interface (mode 1); 2: failure originating at the ceramic–cement interface, progressing into the adhesive resin, and returning to the interface (mode 2); 3: failure starting from an internal flaw (mode 3); 4: failure originating at the ceramic–cement interface and spreading to the adhesive resin (mode 4); 5: failure starting at the ceramic–cement interface and spreading to the adhesive resin to reach the resin composite–cement interface (mode 5) [39]. There was no evidence of cohesive failure in any of the specimens used in this study (Table 4; Figure 1).

### 3.3. Scanning Electron Microscopi (SEM)

From SEM analysis of the surface ceramic (Figure 1A–G), we found that the pattern was mixed with failures where the ceramic surface was partially exposed and partially covered in cement. The resin cement layer is shown in white in the micrographs, while the lithium disilicate surface area is shown in the dark. The ALS group’s exposed ceramic surface area was found to be smaller in the SEM analysis (Figure 1C) and had fewer irregularities than those in the HFS (Figure 1E), SHT2 (Figure 1D), and HF(Figure 1F) groups. The silane heat treatment by Er:YAG laser resulted in additional deterioration and surface imperfections in the ceramic (Figure 1D).

## 4. Discussion

In this study, we investigated the effects of several surface treatments and two silan heat treatment procedures on the binding strength between composite resin and lithium disilicate. The results reject the null hypothesis because silane heat treatment by Er:YAG laser improved the bond strengths between composite resin and lithium disilicate when compared to different surface treatments.Ceramic surface treatment is an important step to improve the strong bond between the ceramic and composite resin [18].

Several studies have found that typical HF treatment followed by silanization considerably boosted the resin bond strength of lithium disilicate ceramics [15,19,24,26]. The effects of HF acid on the morphology of glass ceramics’ surfaces following silane coupling have been thoroughly researched. The chemical reaction between hydrofluoric acid and silica represents the glass phase in lithium-disilicate-based glass-ceramic materials made from hexafluorosilicates. These silicates are removed under running water after HF etching, resulting in micropores that create an asymmetrical roughness on the surface [16,26,40,41]. Furthermore, the impact of silanization on the ceramic surface is indicated by the results of this study. Compared to those ceramics treated with HF and then silanized, specimens treated with HF alone had lower mean TBS values than HFS (*p* < 0.001). By establishing a siloxane link between the ceramic’s inorganic phase and the resin’s cement organic phase, silane agents operate as bridges that boost the surface energy and wettability of the ceramic surface, promoting the resin–ceramic bond strength [19,28,33,34].

Kim et al. [22] investigated the effects of surface treatment on the resin bond strength of various ceramic systems, and revealed that tribochemical coating significantly increased the bond strength of zirconia and alumina-based ceramics. In contrast, a combination of APA and HF etching significantly increased the bond strength of lithium-disilicate-based and feldspathic ceramics.

Bottino et al. [23] studied the effect of surface treatments on the surface morphology of high-alumina and glass-matrix ceramics and concluded that silica coating had no discernible effect on the surface morphology of either ceramic system. Tribochemical silica coating and APA techniques could not be used as alternatives to HF treatment followed by silanization.

Micromechanical retention is commonly achieved via airborne particle abrasion (APA) [17,26]. The lowest mean µTBS value was found in air-abraded and then silanized (ALS) groups in this investigation, which is similar to the findings of some studies [3,20,21]. Silanization alone versus tribochemical silica coating followed by silanization did not significantly differ the results (*p* = 1) according to the findings of this study.

This study aimed to examine how heat treatment affects the resin bond strength of silanized lithium-disilicate-based ceramics. Heat treatment has been shown to significantly increase resin–ceramic bond strength in certain studies [5,13,30,32], whereas it did not in others [3,19,32,33]. In this study, heat treatment with an Er:YAG laser considerably boosted the resin bond strength of silanized lithium disilicate ceramics compared to the other groups. Similar to the present results, a previous study reported that using the Er:YAG laser irradiation before silane treatment can improve a resin’s bond strength to the ceramic surface [18].

Water, alcohol, and other by-products are eliminated from the ceramic surface following silanization before heat treatment, improving the chemical activity of silane coupling agents, and speeding up the condensation process between silicate and silane, boosting resin–ceramic bond strength [19,31,33]. The mean µTBS values of heat-treated, silanized specimens were similar to those for the HFS treatment (*p* < 0.001), which is considerably higher than the other treatment, except for heat treatment with an Er:YAG.

The study’s limitation is that the laboratory study design could not wholly replicate clinical settings. Different outcomes could be obtained at different temperatures, and the varying laser settings could be utilized to affect the alterations in the superficial layers of ceramic surfaces. Furthermore, no additional surface treatment approach that could cause micromechanical roughening and enhance surface energy was used in conjunction with the heat treatment procedures. Perhaps particle abrasions, such as tribochemical silica coating, and subsequent heat treatment after silanization will produce superior bond strength outcomes. To investigate the long-term clinical consequences of post-silanization heat therapy, more in vivo research is needed.

## 5. Conclusions

The mean µTBS value after silane heat treatment by Er:YAG laser increased. The silane heat treatment was similar to HF etching and silane treatment. 

The resin–ceramic bond strength significantly improved after heat treatment and heat treatment can increase the strength of the bond between feldspathic porcelain and composite resin.

## Figures and Tables

**Table 1:** Materials used in the study.

Material	Composition	Manufacturer
IPS e. max Press	Lithium disilicate glass-ceramic	Ivoclar Vivadent, Schaan, Liechtenstein
Tetric N-Ceram	dimethacrylates (19–20 wt %)	Ivoclar Vivadent, Schaan, Liechtenstein
Silane coupling agent	3-glycidoxypropyltrimethoxysilane	Ivoclar Vivadent, Schaan, Liechtenstein
CoJet-System	Sand (CoJet^®®^ Sand): Silicatized sand (particle size 30 μm); Silane (ESPE Sil^®®^): Silane with an attached methacrylic group; and Ethanol Bonding agent (Visio-Bond^®®^): Bisacrylate, Aminodiolmethacrylate, Camphor quinine, Benzyldimethylketale, and Stabilizers	3M ESPE, Seefeld, Germany
Korox 50	Al_2_O_3_ particles	Bego, Bremen, Germany
Porcelain Etch	Hydrofluoric acid	Ultraden Products Inc. South Jordan, UT, USA
Variolink-N	BISGMA, Barium, Glass filler, Di-methacrylates, Pigments, Initiators, Stabilizers, Silica	Ivoclar Vivadent, Schaan, Liechtenstein

**Table 2:** Mean, minimum, maximum bond strength values and standard deviations of groups.

Group	Mean ± SD	Min	Max
**S**	20.12 ^a^ ± 1.71	12	29
**SC**	20.78 ^a^ ± 1.85	14	28
**ALS**	15.62 ^b^ ± 1.79	10	19
**SHT1**	27.46 ^c^ ± 0.97	16	36
**SHT2**	26.74 ^c^ ± 0.82	16	35
**HF**	25.77 ^c^ ± 1.21	20	32
**HFS**	26.06 ^d^ ± 1.41	31	43

No significant differences were found between groups with the same superscript letter.

**Table 3:** Tamhane T2 test results.

Group	CS	ALS	SHT1	SHT2	HF	HFS
**S**	***p*** = 1	***p*** = 0.001	***p*** < 0.001	***p*** < 0.001	***p*** = 0.001	***p*** < 0.001
**SC**		***p*** < 0.001	***p*** < 0.001	***p*** < 0.001	***p*** = 0.008	***p*** < 0.001
**ALS**			***p*** < 0.001	***p*** < 0.001	***p*** < 0.001	***p*** < 0.001
**SHT1**				***p*** < 0.001	***p*** < 0.001	***p*** < 0.001
**SHT2**					***p*** < 0.001	***p*** = 1
**HF**						***p*** < 0.001

**Table 4:** Types of bond failures.

Group	Adhesive *	Cohesive *	Mixed *
S	8 (80)	-	2 (20)
SC	7 (70)	-	3 (30)
ALS	9 (90)	-	1 (10)
SHT1	5 (50)	-	5 (50)
SHT2	6 (60)	-	4 (40)
HF	8 (80)	-	2 (20)
HFS	4 (40)	-	6 (60)
Total	47 (67.1)		23 (32.9)

* n (%).

## Data Availability

The data presented in this study are available upon request from the corresponding author.
